# Cost-effectiveness of AI-enhanced breast cancer screening in Iran: a microsimulation study

**DOI:** 10.1186/s13561-026-00765-5

**Published:** 2026-04-13

**Authors:** Sajjad Dorri Kafrani, Rajabali Daroudi, Morteza Mohammadzadeh

**Affiliations:** 1 Department of Health Management, Policy and Economics, School of Public Health, Tehran University of Medical Sciences, Tehran, Iran; 2 Department of Biostatistics, School of Public Health, Iran University of Medical Sciences, Tehran, Iran

**Keywords:** Microsimulation, Cost Effectiveness, Breast Cancer Screening, Artificial Intelligence

## Abstract

**Background:**

Recognized worldwide as a major health threat and Sustainable Development Goal priority, breast cancer places an especially heavy clinical and economic burden on Iran, making early detection imperative. To guide policy, we assessed the cost-effectiveness of ten mammography-based screening schedules.

**Methods:**

We developed an individual-level microsimulation model simulating 10,000 Iranian women over a 40-year horizon. Screening scenarios varied by interval (annual, biennial, triennial), incorporating mammography alone and two AI-enhanced strategies. Analyses adopted a payer perspective, discounting costs and quality-adjusted life years (QALYs) at 5% annually. Incremental cost-effectiveness ratios (ICERs) were evaluated against a willingness-to-pay (WTP) threshold of USD 4,500 per QALY (Iran’s 2023 GDP per capita). Probabilistic sensitivity analyses (PSA) assessment ensured result robustness.

**Results:**

Compared with no screening, all screening schedules increased QALYs and total costs, while reducing breast-cancer incidence and mortality. Among the alternative tests, annual mammography interpreted by AI and a radiologist was the most cost-effective strategy, with an ICER of USD 4,064 per QALY, demonstrating a high level of cost-effectiveness relative to the next most effective strategy. Probabilistic sensitivity analysis confirmed robustness, with the selected strategy being cost-effective in 52% of simulations.

**Conclusions:**

AI-enhanced mammography is more cost-effective than conventional screening. Concurrent AI-radiologist double reading delivers the greatest value at Iran’s USD 4,500 /QALY threshold, while AI-triage with radiologist confirmation, though less efficient, offers a practical alternative in settings where imaging and biopsy capacity cannot be rapidly expanded.

## Introduction

Breast cancer remains the most frequently diagnosed malignancy and the leading cause of cancer-related mortality among women worldwide. In 2022, approximately 2.3 million women were diagnosed with breast cancer globally, resulting in 670,000 deaths [1]. This underscores the substantial challenge breast cancer poses to global health systems.

In Iran, breast cancer incidence is rising precipitously, accounting for 28.1% of female malignancies [2]. The disease strikes at younger ages compared to Western populations, with a significant prevalence observed among women aged 40–55 [3]. This trend is further corroborated by data from the International Agency for Research on Cancer (IARC), which projects a 63% increase in female breast cancer cases in Iran by 2025 [4].

The increasing incidence imposes a substantial clinical, social, and economic burden on the Iranian healthcare system. This situation underscores the urgent need for effective, resource-aligned screening strategies to enable early detection and improve outcomes. While population-based mammography programs have proven effective in reducing mortality in high-income settings [5] their design often relies on cohort-based models that fail to account for individual-level heterogeneity and contextual constraints, such as Iran’s unique demographic patterns and healthcare infrastructure.

To address this gap, we developed an individual-level microsimulation model to assess the cost-effectiveness of mammography screening strategies tailored to the specific epidemiological and resource contexts of Iran. The study compares conventional mammography with two innovative AI-assisted strategies—concurrent AI and radiologist interpretation, and AI triage with radiologist confirmation. The goal is to identify the most cost-effective approach to breast cancer screening in Iran, providing evidence-based recommendations for policymakers to align health improvements with economic and infrastructural constraints, while also contributing to the broader objectives of the Sustainable Development Goals for equitable and sustainable healthcare.

## Methods

We conducted a payer-perspective cost effectiveness analysis of ten distinct mammography screening scenarios using an individual‐based microsimulation framework. By projecting both clinical outcomes and expenditures over each woman’s lifetime, this person‐level approach captures heterogeneity more precisely than aggregate cohort models. Recognizing that breast cancer tends to arise earlier in Iran than in many Western populations, we defined screening to begin at age 40 and continue at the specified intervals through age 75, in keeping with national life‐expectancy data [6].

We ran each scenario over a 40-year time horizon to fully capture both the immediate impacts of earlier detection and the longer‐term benefits of treatment. Only direct medical costs and health outcomes relevant to payer decisions were included, and all future costs and effects were discounted at an annual rate of 5%. For each screening scenario, we calculated its ICER by comparing it first to the next most effective prior option and then to the no screening scenario.

### Scenarios

In addition to conventional mammography screening at varying intervals (annual, biennial, triennial), we incorporated two advanced strategies involving AI:

1. *Concurrent Mammography and AI Interpretation (strategies sim_mammo_AI_D1–D3)*: In this approach, all mammograms are independently assessed by both a radiologist and an AI algorithm. A case is considered screen-positive if either the AI or the radiologist (or both) flag it as positive. This dual-review mechanism aims to increase sensitivity by leveraging both human expertise and machine-based detection.
2. *AI-Triage with Radiologist Confirmation (strategies sim_mammo_AIR_D1–D3)*: In this strategy, mammograms are first evaluated by AI. Only those identified as positive by the AI are then reviewed by a radiologist for confirmation. A case is considered screen-positive only if confirmed by the radiologist, which may improve specificity and reduce false positives.

The ten screening scenarios, identified by their model codes and defined by starting age, ending age, and screening intervals, are summarized in Table 1.

**Table 1:** Screening scenarios

Strategy	Definition
*sim_noscreening*	No screening intervention (reference scenario).
*sim_mammo_D1*	Annual mammography beginning at age 40 and continuing through age 75.
*sim_mammo_D2*	Biennial mammography beginning at age 40 and continuing through age 75.
*sim_mammo_D3*	Triennial mammography beginning at age 40 and continuing through age 75.
*sim_mammo_AI_D1*	Annual mammography with concurrent interpretation by AI and a radiologist, beginning at age 40 and continuing through age 75. (Concurrent Mammography and AI Interpretation)
*sim_mammo_AI_D2*	Biennial mammography with concurrent interpretation by AI and a radiologist, beginning at age 40 and continuing through age 75. (Concurrent Mammography and AI Interpretation)
*sim_mammo_AI_D3*	Triennial mammography with concurrent interpretation by AI and a radiologist, beginning at age 40 and continuing through age 75. (Concurrent Mammography and AI Interpretation)
*sim_mammo_AIR_D1*	Annual mammography interpreted by AI with radiologist confirmation, beginning at age 40 and continuing through age 75. (AI-Triage with Radiologist Confirmation)
*sim_mammo_AIR_D2*	Biennial mammography interpreted by AI with radiologist confirmation, beginning at age 40 and continuing through age 75. (AI-Triage with Radiologist Confirmation)
*sim_mammo_AIR_D3*	Triennial mammography interpreted by AI with radiologist confirmation, beginning at age 40 and continuing through age 75. (AI-Triage with Radiologist Confirmation)

### Model framework

In this model, each woman progresses through a set of mutually exclusive health states in annual cycles, reflecting the natural history of breast cancer. These states include: Healthy (no detectable disease), Ductal Carcinoma in Situ (DCIS), Invasive Cancer (Stages I–IV), Post-Treatment Survivor, Death from Breast Cancer, and Death from Other Causes.

**Fig. 1:**
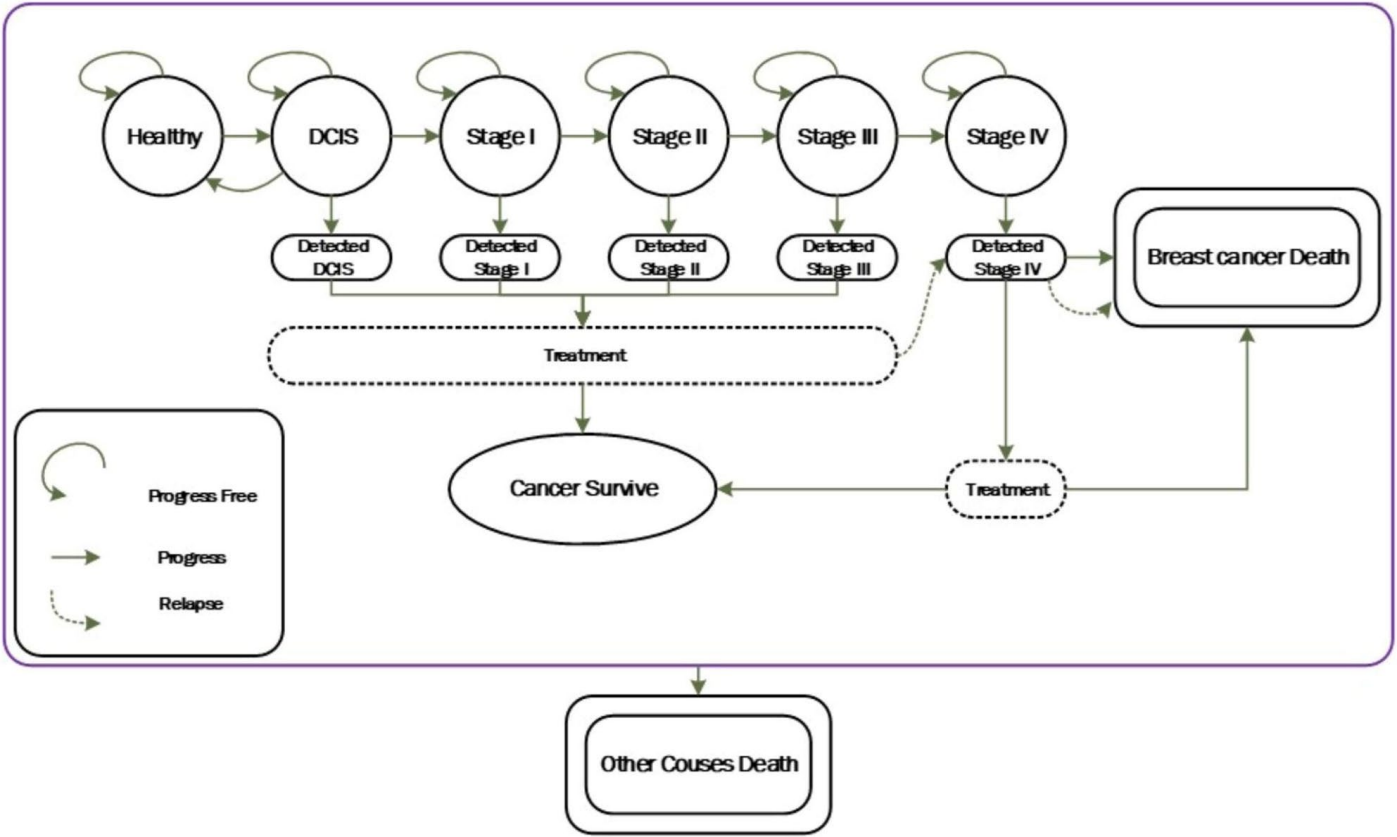
Natural history of breast cancer

Figure 1 illustrates how, over each one-year interval, a woman’s disease can advance (or, in DCIS, occasionally regress), and how both cancer‐specific and background mortality apply. The core assumptions are:

1. A healthy individual may develop DCIS.
2. A proportion of DCIS cases resolve without progressing.
3. At any point, each woman faces an all-cause mortality risk appropriate to her current state and age.
4. Only those whose cancer advances to Stage IV can die of breast cancer.

Transition probabilities—governing progression, regression, and death—were drawn from peer-reviewed literature and locally calibrated incidence and survival data. By simulating thousands of individual life courses, this framework captures the heterogeneity in progression and allows direct evaluation of how earlier screening shifts outcomes.

### Model implementation in R

**Fig. 2:**
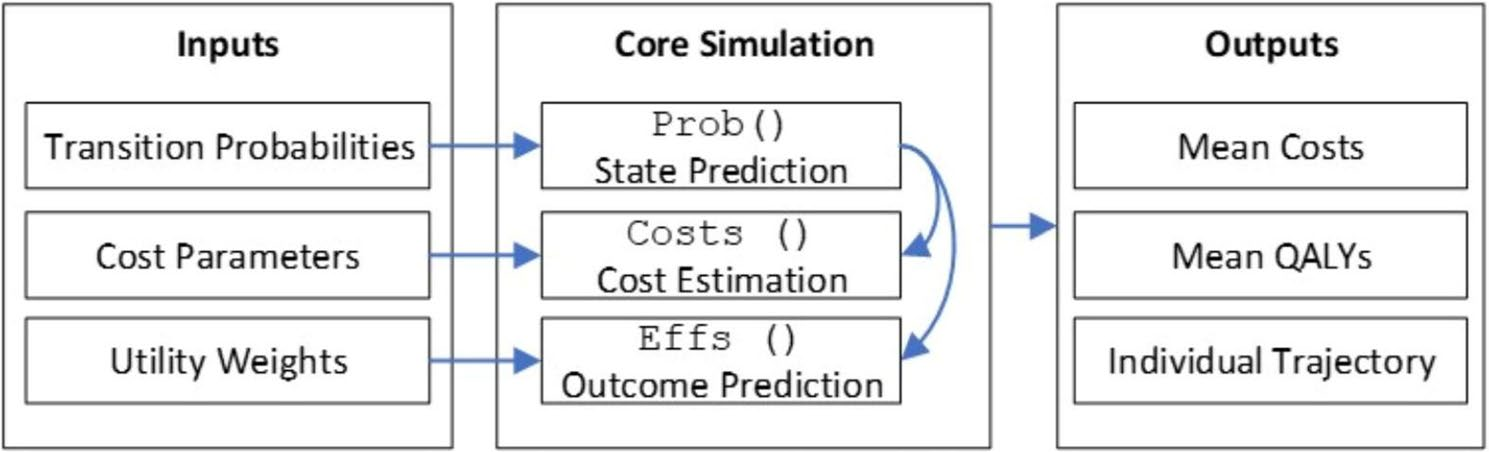
Workflow of the microsimulation framework in R

Our microsimulation is coded in R and organized into three sequential modules (Fig. 2): data preparation, core simulation, and results extraction. Once all input parameters are loaded, the driver function *MicroSim()* launches the simulation. During each annual cycle for every woman, *MicroSim()* calls three nested functions:

- *Probs*: samples the next health state based on the current state, cycle index, and screening status.
- *Costs*: assigns direct medical expenditures—biopsy; stage-specific treatment in year 1 and years 2–5; mammography and diagnostic follow-up; and routine survivor care—adding screening costs only when a scheduled mammogram occurs.
- *Effs*: assigns a health-related quality of life (HRQoL) value to each state and computes QALYs accrued during that cycle.

These outputs are stored in state, cost, and utility matrices, with reproducibility ensured by fixed random seeds. At the end of the 40-cycle horizon, costs and QALYs are discounted at 5% per annum and summed across cycles to obtain present-value totals for each individual. Cohort averages of discounted costs and QALYs for each screening strategy then feed directly into ICERs calculations. Our implementation choices and parameter distributions align with established recommendations for decision modeling in health economic evaluation [7, 8].

### Model parameters

#### Transition probabilities

The transition probabilities between different health states in the microsimulation model were established based on data from well-established studies and credible international and national sources. Incidence and mortality rates for breast cancer across various age groups were obtained from the IARC [9]. Stage-specific survival rates during the first five years after diagnosis were calculated using national survival data. For those surviving beyond this period, mortality rates were adjusted using age-specific mortality rates derived from life tables of the general population [10].

Due to the lack of specific data regarding the transition from a healthy state to DCIS, a calibration process was employed to estimate these probabilities, ensuring they aligned with observed disease trends. While some DCIS cases may resolve naturally, others progress to Stage I breast cancer. The transition probabilities from DCIS to early-stage cancer, as well as its further progression to advanced stages, were generated by randomly sampling derived from distributions fit to published progression data. The distribution of Iranian breast cancer patients across different stages provided the foundation for estimating initial probabilities [11–13].

#### Test characteristics

The diagnostic accuracy of the tests was modeled based on mammography, AI and biopsy, considering patient cancer stage to ensure these variables were accurately reflected in the model. Due to the lack of reliable national studies in Iran, sensitivity and specificity estimates were derived from high-quality international research. In cases where stage-specific sensitivity and specificity values were unavailable, estimates were adjusted using a data-refinement approach. This involved adjusting pooled sensitivity values by incorporating stage distributions from other studies to ensure consistency with observed patterns, providing a more accurate representation of test performance across various disease stages [14, 15].

For biopsy, considered as the confirmatory diagnostic test, it was assumed that its sensitivity would be 100%. Further details regarding the data sources can be found in Table 2.

**Table 2:** Model parameters

Annual age-specific incidence of invasive breast cancer								Source
Age Group			Baseline value					Model calibration
40–44			0.0004854					
45–49			0.0005101					
50–54			0.0017720					
55–59			0.0017103					
60–64			0.0016956					
65–69			0.0025666					
70–74			0.0017920					
75–79			0.0006337					

Annual all-cause mortality by age group (females)								
Age Group			Baseline value					[10]
40–44			0.001061					
45–49			0.001607					
50–54			0.002597					
55–59			0.004357					
60–64			0.007751					
65–69			0.012888					
70–74			0.022476					
75–79			0.043894					

**Relapse Rate**								
**Year**	**Stage**	**Baseline value**						**Source**
During first Year	**I**	0.04310727						[16, 17]
	**II**	0.2099324						
	**III**	0.524831						
From year 2 to 5	**I**	0.1193797						
	**II**	0.1524264						
	**III**	0.06317258						

**Parameter**						**Baseline value**		**Source**
Mortality of stage IV female breast cancer during the first year after diagnosis						0.742725		[16, 17]
Mortality of stage IV female breast cancer during years 2 to 5 after diagnosis (Yearly)						0.2642997		

**Annual transition probabilities**								
**Parameter**			**Baseline value**	**Range**			**Distribution**	**Source**
				**Low**	**Up**			
DCIS to Stage (I)			0.21	0.16	0.29		Beta	[18–20]
Stage (I) to Stage (II)			0.24	0.19	0.29		Beta	
Stage (II) to Stage (III)			0.29	0.23	0.35		Beta	
Stage (III) to Stage (IV)			0.50	0.40	0.60		Beta	
DCIS to Healthy			0.25	0.20	0.30		Beta	
Diagnosed from Stage (I)			0.025	0.02	0.03		Beta	[21]
Diagnosed from Stage (II)			0.22	0.18	0.26		Beta	
Diagnosed from Stage (III)			0.55	0.44	0.66		Beta	
False-positive to biopsy			1.00	0.95	1.00		Beta	Expert’s Opinion
Diagnostic test characteristics								
Stage-specific sensitivity and specificity of mammography								
DCIS			0.40	0.38	0.42		Beta	[14, 15]
Stage (I)			0.77	0.74	0.82		Beta	
Stage (II)			0.94	0.90	1.00		Beta	
Stage (III)			0.99	0.95	1.00		Beta	
specificity			0.96	0.95	0.97		Beta	
Stage-specific sensitivity and specificity of concurrent mammography and AI interpretation								
DCIS			0.45	0.36	0.54		Beta	
Stage (I)			0.89	0.71	1.00		Beta	
Stage (II)			0.99	0.95	1.00		Beta	
Stage (III)			0.99	0.95	1.00		Beta	
specificity			0.93	0.90	1.00		Beta	
Stage-specific sensitivity and specificity of AI-triage with radiologist confirmation								
DCIS			0.17	0.13	0.20		Beta	
Stage (I)			0.63	0.50	0.75		Beta	
Stage (II)			0.93	0.74	1.00		Beta	
Stage (III)			0.99	0.95	1.00		Beta	
specificity			0.966	0.95	1.00		Beta	
Biopsy								
Biopsy sensitivity			1.00	0.95	1.00		Beta	Model Assumption
Biopsy specificity			1.00	0.95	1.00		Beta	
Utility values								
Quality of life								
Healthy								
< 40			1.00					[22]
40–49			0.75					
50–59			0.71					
60–69			0.68					
70–90			0.65					
DCIS			0.61	0.52	0.70		Log-normal	[22, 23]
Stage (I)			0.63	0.62	0.65		Log-normal	
Stage (II)			0.63	0.63	0.64		Log-normal	
Stage (III)			0.62	0.61	0.64		Log-normal	
Stage (IV)			0.55	0.51	0.60		Log-normal	
Disutility of false-positive anxiety			0.00	0.00	0.01		Log-normal	Expert’s Opinion
Disutility of biopsy			0.00	0.00	0.01		Log-normal	
Cost and QALY discounting rate			0.05	0.04	0.06		Beta	
Costs (US$)								
Diagnosis								
Mammography			7.71	2.14	14.98		Gamma	Tariffs
Biopsy			21.67	1.66	28.68		Gamma	
Coordination and follow up ^a^			0.68	0.55	0.82		Gamma	TDABC ^b^
Treatment and follow-up during the first year								
DCIS			409	327	491		Gamma	[24–26]
Stage (I)			698	559	838		Gamma	
Stage (II)			2406	1925	2887		Gamma	
Stage (III)			3203	2562	3844		Gamma	
Stage (IV)			4136	3309	4963		Gamma	
Treatment and follow up during years 2 to 5 (Yearly)								
DCIS			65	52	78		Gamma	[24–26]
Stage (I)			111	89	133		Gamma	
Stage (II)			478	383	574		Gamma	
Stage (III)			1008	807	1210		Gamma	
Stage (IV)			3178	2542	3814		Gamma	
The distribution of individuals at the commencement of the modeling process								
Stage			Distribution (%)					
Stage (I)			11					[11–13]
Stage (II)			51					
Stage (III)			31					
Stage (IV)			7					

**Other inputs**								
**Description**			**Name in model**		**Value**			**Source**
The number of individuals			n.i		10,00			Model Assumption
The number of model cycles			n.t		40			
Discount costs			d.c		0.05			
Discount effects			d.e		0.05			
Start Screening			start_mammo		40			
Intake Rate			intake_rate		0.8			Expert’s Opinion

^a^Call for mammography and follow-up for further actions if needed

^b^Time-Driven Activity-Based Costing

### Cost parameters

All expenditures included in this analysis were tallied from the perspective of the payer, incorporating only those outlays directly tied to medical care. Screening-related fees-for example, mammograms and core-needle biopsies-were taken from the official 2023 diagnostic tariff schedule issued by Iran’s Ministry of Health and Medical Education. For patients confirmed with breast cancer, we then captured the full first‐year treatment bill and all subsequent disease‐management costs over the following four years-including clinic visits, imaging, laboratory work, medications, and complication management-stratifying these sums by both stage (I through IV) and year of follow-up.

The development costs of AI-assisted tools were estimated based on literature reviews and expert input. These costs include research and development (R&D) expenses, software engineering, and integration of AI algorithms into existing screening workflows. Additional costs for training healthcare professionals to use these AI tools were also considered, as well as the ongoing maintenance and updates required to ensure the tool’s effectiveness over time.

As previously described in the Scenarios section, our model includes two distinct AI-assisted mammography workflows. Both workflows incur the same fixed costs for AI software development, integration, training, and maintenance, estimated from literature reviews and expert input. The variable cost of radiologist reading is applied per interpreted mammogram in the concurrent strategy and per triage-positive mammogram in the AI-triage strategy. Downstream costs of false positives (additional biopsies, patient anxiety) and false negatives (delayed treatment, advanced-stage cancer costs) are incorporated through stage-specific treatment costs.

The time and resources necessary to coordinate care after a positive screen result—such as secretarial work, scheduling, and follow-up calls—were costed via a Time-Driven, Activity-Based Costing (TDABC) exercise. All Rials-denominated figures were converted to U.S. dollars at the prevailing 2023 exchange rate (490,000 IRR = 1 USD), and then updated to current values by applying the official inflation indices published by the Central Bank.

### Health utilities

We used QALYs to quantify the impact of screening scenarios, capturing both survival duration and health-related quality of life. Each health state was assigned a utility weight—from 1.0 (perfect health) down to 0.0 (death)—and a patient’s time in that state was multiplied by its corresponding utility to yield QALYs. Baseline utility norms, segmented by age bracket, were drawn from domestic population surveys to reflect the general Iranian health profile [22]. Cancer-stage utilities were then informed by published international research and locally adjusted to account for Iran‐specific care experiences and outcomes (Table 2).

### Modeling screening harms

To comprehensively account for screening-related harms, the model incorporated specific parameters for false-positive results and overdiagnosis. The costs for individuals undergoing biopsy were specifically obtained from our TDABC analysis. Furthermore, Overdiagnosis was defined as the detection of a Ductal Carcinoma in Situ (DCIS) that would never have progressed to invasive cancer during a woman’s lifetime. In the model, a proportion of DCIS cases transition to a “healthy” state, representing non-progressive lesions. The costs lost associated with diagnosing and treating these non-progressive DCIS cases were fully captured, thereby incorporating the burden of overdiagnosis into the incremental cost-effectiveness calculations.

### Model calibration

We tuned the model to match empirical age‑specific breast cancer incidence figures obtained from the IARC repository, widely regarded as the most comprehensive source for Iran’s cancer statistics. Predicted rates—grouped in five‑year age bands—were compared against the IARC data, and discrepancies were resolved iteratively through parameter adjustments until the simulated and reported rates aligned closely. This calibration to Iranian age‑specific incidence data ensures that the younger age‑at‑diagnosis pattern observed in the country is accurately reflected, thereby incorporating the aggregate effect of local risk factors (including genetic, reproductive, and lifestyle influences) without requiring explicit modeling of each individual determinant.

### Analysis

We evaluated ten breast cancer screening scenarios by calculating their ICERs. Each approach was compared stepwise against the next most effective option based on differences in cost and health benefit; any scenario found to be more expensive yet less effective or yielding a higher ICER than a more favorable alternative, was removed from consideration as “dominated.” We judged scenarios to be cost-effective if their ICER fell below Iran’s 2023 willingness‐to‐pay threshold of USD 4,500 per QALY, equal to one times the nation’s per capita GDP. To reflect the time value of money and health benefits, both costs and outcomes were discounted at 5% annually. Analyses were performed in R 4.5.0 (RStudio 2024.12.1.563) on a Dell Inspiron N5050 with an Intel Celeron dual-core 1.6 GHz CPU, 6 GB RAM, and a 500 GB SATA drive.

### Sensitivity analysis

#### Probabilistic sensitivity analysis

To assess the robustness of our cost-effectiveness findings and ensure the precision of our individual‐level microsimulation, we first determined an appropriate cohort size by incrementally increasing the number of virtual patients and tracking the resulting variance in ICERs. We found that variability of simulation results diminished markedly as the sample grew, and beyond approximately 10,000 simulated women further increases yielded only marginal reductions in fluctuation.

Having identified this stabilization point, we then performed a probabilistic sensitivity analysis by varying all model inputs simultaneously according to predefined probability distributions. We ran the full model 1000 times with 10,000 individuals each, sampling from each input’s distribution, to generate a comprehensive distribution of ICERs. This approach both ensured that our estimates are precise and quantified the impact of parameter uncertainty on our conclusions.

### Deterministic sensitivity analysis

To examine the robustness of the model’s cost‑effectiveness findings, a deterministic scenario‑based sensitivity analysis was conducted. This analysis focused on key parameters: AI‑assisted diagnostic accuracy, the annual discount rate, the probability of biopsy following a false‑positive screening result, the probability of biopsy following a false-positive screening result, the anxiety associated with false-positive mammography results, and the per‑person AI implementation cost.

For each parameter, three plausible scenarios-a base‑case, an optimistic, and a conservative scenario-were defined to span the range of credible values. The scenario values were informed by published literature and empirical screening data where available. The microsimulation model was re‑run under each scenario while holding all other inputs at their base‑case levels, allowing the isolated impact of each parameter on total costs, total QALYs, and the ICER to be assessed.

## Results

### Model calibration

**Fig. 3:**
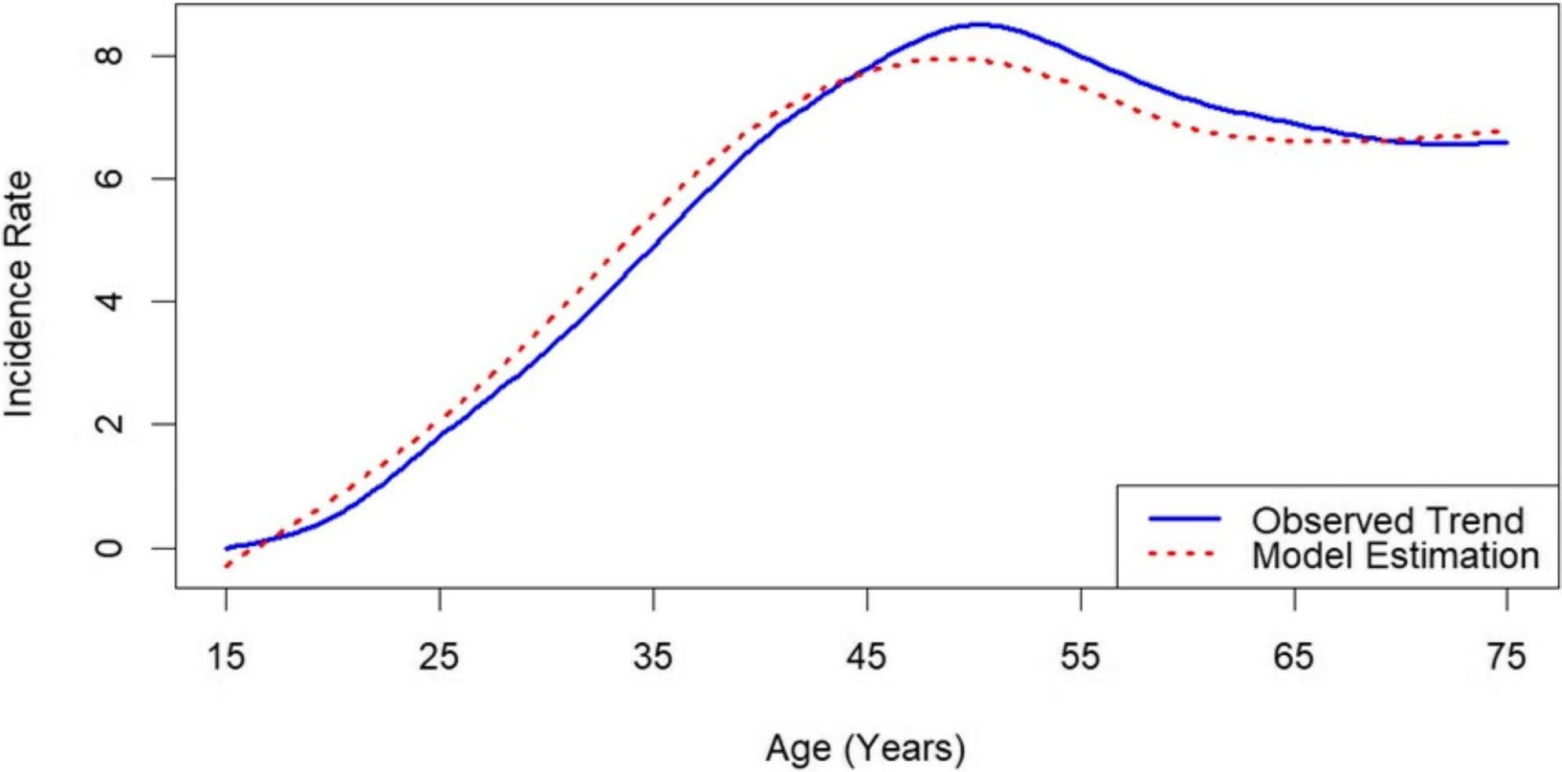
Comparison of age psecific incidence rate

We calibrated our microsimulation against age-specific, sex-standardized registry data to ensure that it accurately reproduces real-world breast-cancer incidence patterns. Across all age groups, the model’s predicted and observed rates match closely, yielding a root-mean-square deviation of 0.80, just 15.1% of the mean observed rate, demonstrating good concordance with empirical data (Fig. 3). This close fit indicates that the chosen transition probabilities, sojourn-time distributions, and competing-risk adjustments collectively yield a realistic disease trajectory. Taken together, the calibration results confirm that the model faithfully captures the underlying epidemiology of breast cancer in the study population and can therefore serve as a reliable baseline for subsequent counterfactual analyses.

### Base-case results

**Fig. 4:**
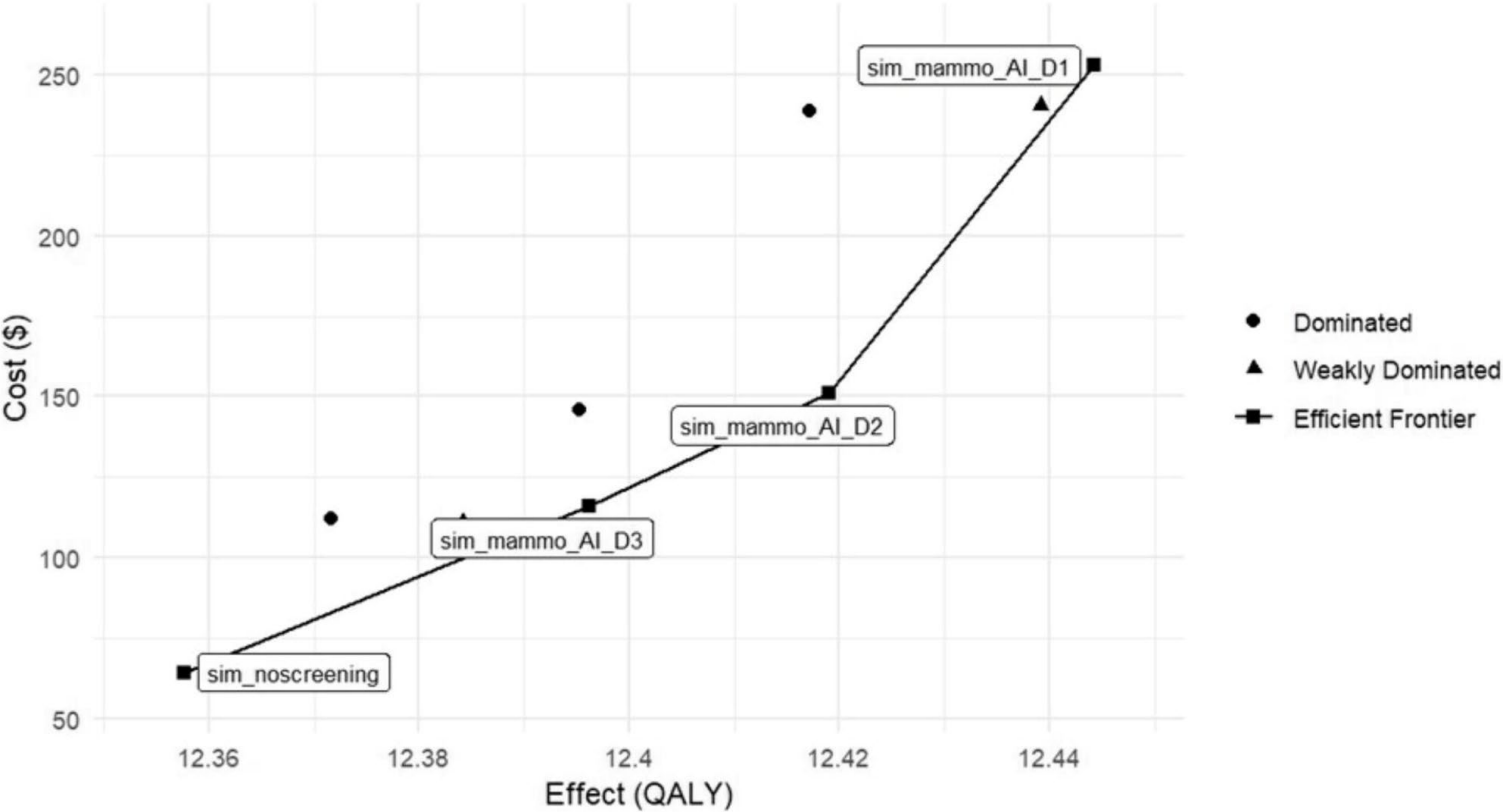
Cost-effectiveness plan of breast cancer screening strategies

In our primary analysis, we compared each of the nine mammography-based screening schedules both to the “no screening” baseline and, incrementally, against the most effective preceding strategy to assess their relative cost‐effectiveness (Tables 3 and 4). Among these, four strategies formed the cost‐effectiveness frontier-meaning they delivered the greatest health benefits (measured in QALYs) for their respective additional costs and are thus considered economically attractive choices (Fig. 4).

**Table 3:** Cost-effectiveness analysis results

Scenario	Cost	Effect	Inc_Cost	Inc_Effect	ICER	Status
sim_noscreening	64.41	12.3577				ND
sim_mammo_AI_D3	115.91	12.3962	51.50	0.04	1,337	ND
sim_mammo_AI_D2	150.93	12.4191	35.02	0.02	1,533	ND
sim_mammo_AI_D1	253.03	12.4442	102.10	0.03	4,064	ND

*ND* Not Dominated, *Inc* Incremental

**Table 4:** ICER of screening strategies vs. no screening

Scenario	Cost	Effect	ICER	Status
sim_noscreening	64.41	12.3577		ND
sim_mammo_D3	110.92	12.3842	1,754	ED
sim_mammo_AIR_D3	112.28	12.3717	3,423	D
sim_mammo_AI_D3	115.91	12.3962	1,337	ND
sim_mammo_D2	143.57	12.4110	1,486	ED
sim_mammo_AIR_D2	145.97	12.3952	2,174	D
sim_mammo_AI_D2	150.93	12.4191	1,410	ND
sim_mammo_AIR_D1	238.74	12.4172	2,929	D
sim_mammo_D1	240.51	12.4392	2,161	ED
sim_mammo_AI_D1	253.03	12.4442	2,180	ND

*ND *Not Dominated, *D* Dominated, *ED* Extended Dominated, *ICER* Incremental Cost-Effectiveness Ratio

All nine screening scenarios yielded improvements in QALY over the no-screening option, but these health gains came at the price of increased per‐person expenditures (Table 4). When judged against a willingness‐to‐pay ceiling of US $4,500 per QALY—equal to Iran’s GDP per capita in 2023- the strategy of annual mammography interpreted concurrently by both AI and a radiologist (sim_mammo_AI_D1) emerged as the standout, offering the best balance of cost and benefit.

In the absence of screening, the model predicts 271 new cases and 139 deaths per 10,000 women. All screening strategies yield substantial reductions in mortality (37%–71%) and incidence (19%–54%) relative to no screening. Annual concurrent interpretation (sim_mammo_AI_D1) achieves the largest absolute reduction in deaths (− 75%, 40 vs. 139 per 10,000) and new cases (− 54%, 123 vs. 271 per 10,000), highlighting the value of AI-assisted techniques in enhancing early detection.

Incorporating radiologist confirmation of only AI-positive cases (sim_mammo_AIR_D*) results in slightly higher mortality (68–87 per 10,000) and incidence (211–219 per 100,000) compared to concurrent interpretation of AI and radiologist, reflecting the trade-off between the level of expenditure and gained health benefits (Table 5).

**Table 5:** Comparison of breast cancer incidence and mortality across screening strategies

Strategy	Number of Breast Cancer Death per 10,000	Number of Breast Cancer Incidence per 10,000
noscreening	139	271
mammo_AIR_D3	87	219
mammo_AIR_D2	72	213
mammo_AIR_D1	68	211
mammo_D3	71	179
mammo_AI_D3	64	176
mammo_AI_D2	59	165
mammo_D2	60	160
mammo_D1	42	139
mammo_AI_D1	40	123

### Sensitivity analysis

#### Probabilistic sensitivity analysis

**Fig. 5:**
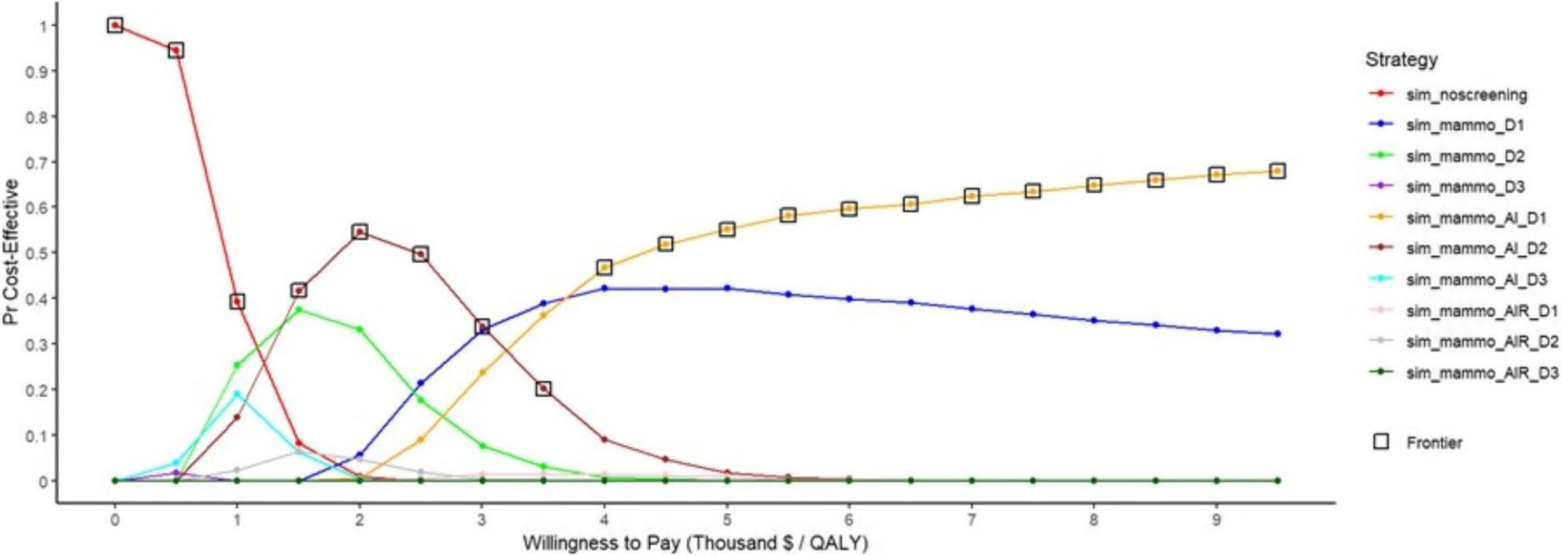
Cost effectiveness acceptability frontier of breast cancer screening strategies

The results of the probabilistic sensitivity analysis, derived from a Monte Carlo simulation with 1,000 iterations, are presented in the cost-effectiveness acceptability frontier (see Fig. 5). This curve illustrates that as the willingness to pay increases, the probability of the no-screening option being cost-effective decreases, while the cost-effectiveness of the mammography strategy enhanced with AI rises for both biennial and annual screening intervals. Notably, when the willingness to pay threshold exceeds $4,000, the annual screening strategy becomes the most cost-effective option with a high probability. The probabilistic sensitivity analysis findings, corroborated by the Monte Carlo simulation, confirm that the base case results are both highly probable and robust.

**Fig. 6:**
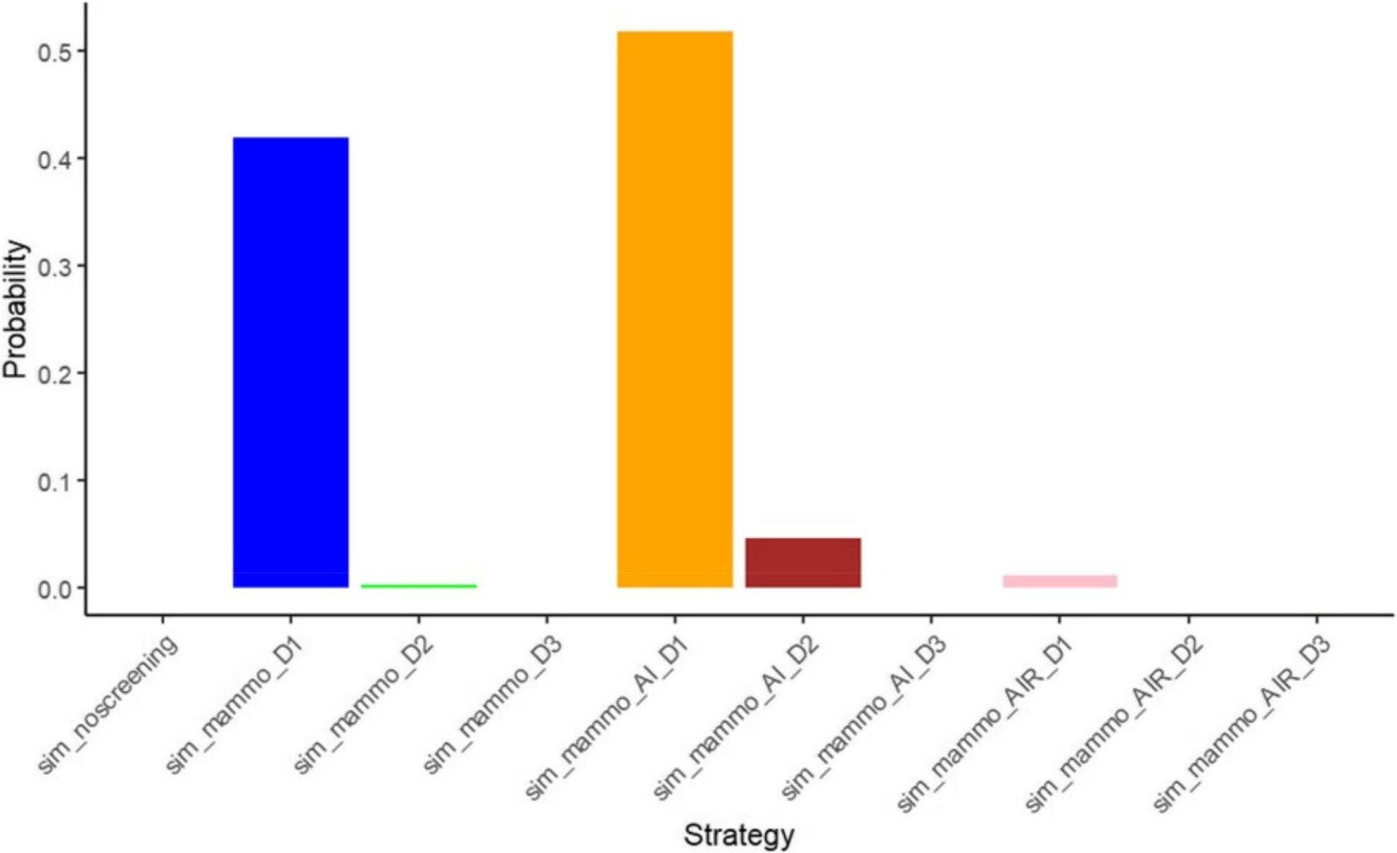
Monte Carlo strategy selection (WTP = 4500)

The strategy-selection diagram (Fig. 6) corroborates these findings. At a WTP ceiling of US $4,500 per QALY, probabilistic sensitivity analysis based on 1,000 Monte-Carlo iterations shows that sim_mammo_AI_D1 is the cost-effective choice in 52% of simulations. The likelihood for sim_mammo_D1 to be optimal is 42%, whereas sim_mammo_AI_D2 attains this status in only 4% of runs. Collectively, these results underscore the predominance of sim_mammo_AI_D1 at the specified economic threshold.

### Deterministic sensitivity analysis

**Fig. 7:**
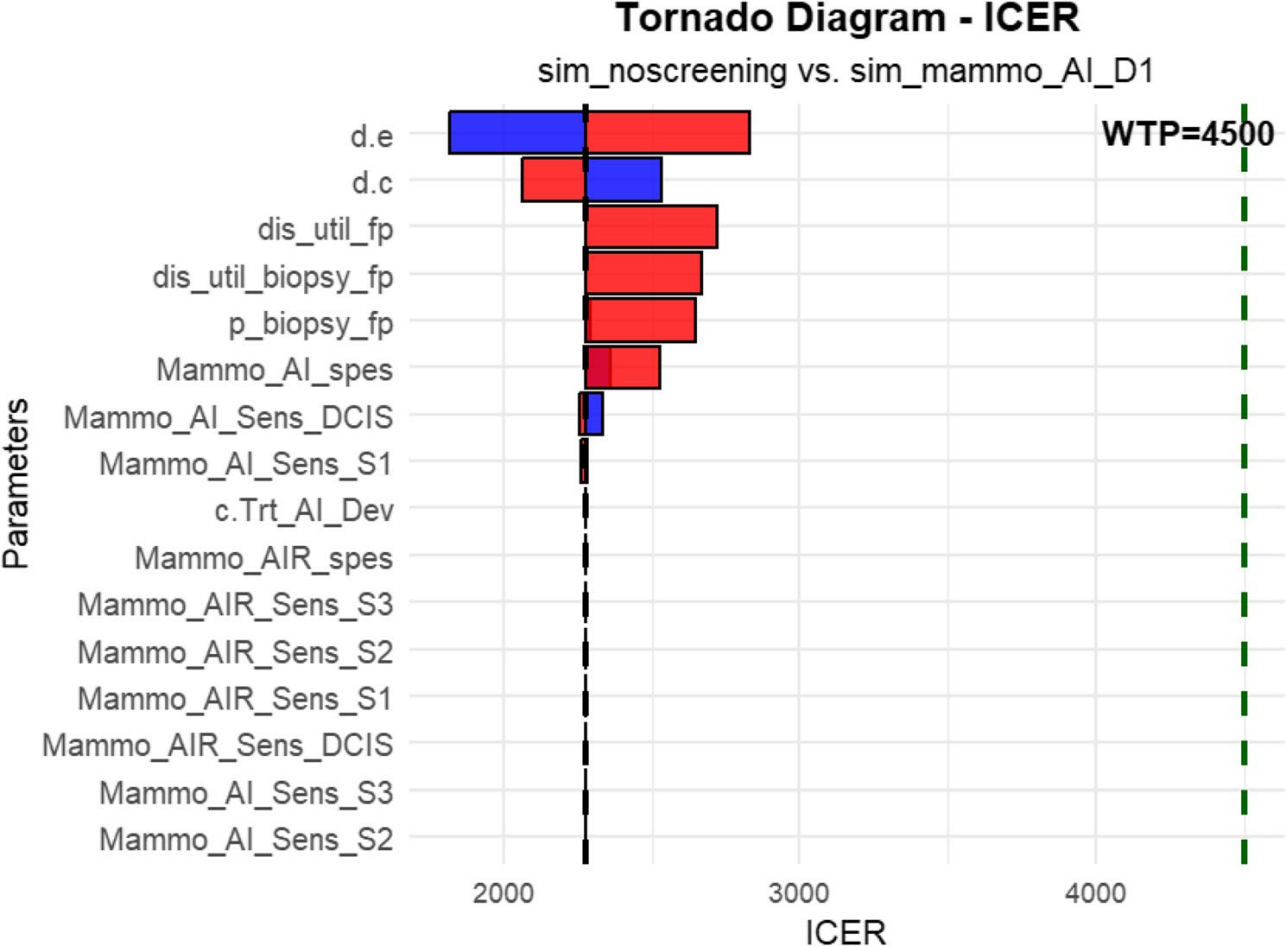
Tornado diagram for one-way sensitivity analysis

Most of the parameters in the one-way sensitivity analysis had a limited effect on the results. The variables that influenced the model outcomes the most were discount rates and disutility of false positive while parameters related to mammography AI sensitivity showed relatively smaller effects (Fig. 7).

**Fig. 8:**
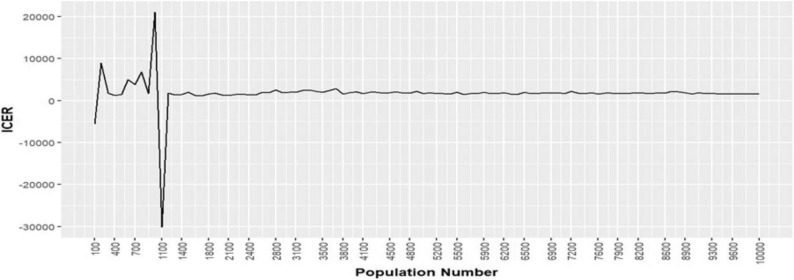
Convergence of ICER estimates as microsimulation cohort size increases

We explored how the number of simulated individuals affected the stability of our cost-effectiveness outcomes. Beginning with a cohort of 100 women, we observed relatively large fluctuations in key metrics (ICERs) as the sample was increased (Fig. 8). As we scaled up to 8,500 simulated individuals, however, these fluctuations diminished markedly—indicating that stochastic noise was being averaged out and our estimates were converging. By the time we reached 10,000 simulated women, virtually no further changes in results were detectable and ICERs lay within a narrow band around their long-run means.

## Discussion

Our individual-level microsimulation model provides a robust framework for evaluating breast cancer screening strategies in Iran, identifying annual mammography with concurrent AI and radiologist interpretation (sim_mammo_AI_D1) as the most cost-effective approach at a WTP threshold of USD 4,500 per QALY, equivalent to Iran’s 2023 per capita GDP. Although an annual schedule entails higher screening volume than biennial or triennial alternatives, the additional health gains achieved through earlier-stage detection and the resultant downstream savings in treatment costs more than offset the incremental program expenses. By leveraging AI to bolster diagnostic accuracy and shorten reading times, this intensified schedule remains operationally feasible even in settings where radiologist availability and biopsy capacity are constrained. Consequently, scaling up to annual AI-assisted mammography offers the greatest value for money within Iran’s National Cancer Control Program, provided that capacity-building efforts—particularly in rural provinces—accompany its phased implementation.

Building on individual-level microsimulation studies like the UK’s MISCAN–Fadia comparisons of annual versus biennial and triennial intervals [27] and Ahern et al.’s analysis of MRI‐augmented screening in high‐risk women [28]—as well as Hill et al.’s risk‐stratified UK protocols [29]— our work extends the literature by evaluating a comprehensive set of mammography-based strategies, including both conventional and AI-assisted approaches, to construct a full cost-effectiveness frontier. Unlike models calibrated to high‐income settings, we tailor ours to Iran’s epidemiology, cost structures, and a GDP‐based willingness‐to‐pay threshold, focus on simple strategies for the general population rather than high‐risk or multimodal approaches, and embed extensive probabilistic sensitivity and sample‐size analyses.

The incorporation of AI-assisted strategies marks a clear advance over traditional mammography programs. Our analysis shows that annual AI-assisted screening (sim_mammo_AI_D1) now delivers the most favorable cost-effectiveness profile, with an ICER well below the USD 4,500 per-QALY WTP threshold. Compared with conventional annual mammography without AI (sim_mammo_D1), this hybrid approach detects more early-stage cancers, reduces false-positive callbacks, and ultimately generates larger QALY gains at a lower incremental cost.

Although AI triage followed by radiologist confirmation fits Iran’s workforce and infrastructure constraints—because the algorithm screens out obviously normal studies and reserves specialist time—this operational advantage comes at a clinical and economic cost. The triage threshold lowers overall test sensitivity, leading to fewer early cancer detections and markedly smaller QALY gains. In our model, the savings in interpretation time do not offset these losses, so the incremental cost-effectiveness of the triage pathway improves only marginally—and remains inferior to that of concurrent AI-plus-radiologist double reading (sim_mammo_AI_D1). Hence, at the current WTP ceiling of USD 4,500 per QALY, concurrent double reading is still the economically preferred option; AI triage should be reserved for settings where rapid expansion of radiological capacity is not feasible.

From a policy standpoint AI-assisted mammography could fundamentally reshape Iran’s breast-cancer-screening landscape. Compared with the current opportunistic model—reaching fewer than 30% of eligible women even in cities with populations above 50 000—an organized yearly program would dramatically increase the volume of screening examinations. While AI can offset some of this added workload by shortening reading times and lowering recall rates, a successful rollout still demands targeted investments: procuring AI-ready digital mammography units, delivering hands-on training for radiologists and technologists, and building robust patient-navigation pathways to guarantee timely diagnostic follow-up.

Our study’s strengths lie in its rigorous methodology: calibration to Iran’s cancer registry data ensures epidemiological accuracy, while PSA confirm the robustness of our cost-effectiveness estimates across a range of uncertainties. The model’s ability to simulate 10,000 individual life courses captures the full spectrum of disease trajectories, providing a more nuanced evaluation than traditional cohort models.

Nonetheless, our analysis has limitations. First, we focused solely on mammography-based pathways, excluding emerging modalities such as automated ultrasound or molecular assays, which could further enhance early detection. Future iterations of our model could incorporate these technologies by specifying their stage-specific performance and costs. Second, systemic challenges—such as limited diagnostic capacity, workforce shortages, and regional disparities—may hinder nationwide scale-up. Addressing these requires comprehensive capacity assessments and investments in infrastructure, particularly in rural areas.

Third, our model assumed a homogeneous population and did not stratify outcomes by genetic predisposition, socioeconomic status, or ethnicity due to the lack of nationally representative data in Iran. Consequently, the cost‑effectiveness estimates reflect average effects for the general population of Iranian women aged 40–79. Future availability of reliable subgroup data would allow extending the framework to evaluate risk‑stratified screening strategies. Fourth, while the model captures the younger age-at-diagnosis pattern observed in Iran through calibration to local incidence data, it does not explicitly parameterize specific etiological factors (e.g., dietary patterns, genetic predispositions) that may underlie this pattern. This omission limits the direct extrapolation of our results to populations with substantially different risk profiles. For such applications, recalibration of the model using local age-specific incidence data is necessary to ensure that the simulated epidemiological profile aligns with the target population.

Our findings also contribute to the global pursuit of Sustainable Development Goal 3, which calls for reducing premature mortality from non-communicable diseases. By identifying a cost-effective, scalable screening strategy, our study provides a blueprint for aligning Iran’s breast cancer control efforts with global health priorities.

Future research should explore additional innovations, such as tomosynthesis or risk-stratified protocols, and incorporate real-world data on AI performance and implementation costs in Iran. Moreover, longitudinal studies evaluating the feasibility of scaling up AI-assisted screening in resource-limited settings will be critical to translating our findings into practice.

## Conclusion

Our individual-level microsimulation indicates that annual mammography interpreted concurrently by an AI system and a radiologist (sim_mammo_AI_D1) offers the greatest value at the USD 4,500 per-QALY threshold, detecting more early-stage cancers, preventing costly advanced disease, and generating the largest net QALY gain despite its higher screening volume. In contrast, an alternative AI-enabled workflow—AI triage followed by radiologist confirmation—eases pressure on the limited specialist workforce but sacrifices sensitivity and therefore cost-effectiveness, making it suitable only as a temporary solution in provinces that cannot quickly expand imaging and biopsy capacity.

Implementing the strategy requires AI-ready mammography units, and expanded training for imaging staff, supplemented by mobile and teleradiology services with strong patient navigation to ensure timely care; a national registry tracking AI performance, interval cancers, and costs will support continuous improvement. Future work should evaluate risk-based screening, test tomosynthesis and automated ultrasound, and gather real-world cost data—steps that will help establish a sustainable, equitable program aligned with SDG 3 and inform other resource-constrained countries.

## Data Availability

Individual participant data will not be available. Other requests will be considered upon receipt by the corresponding author.

## Abbreviations

QALYs: Quality-Adjusted Life Years
ICERs: Incremental Cost-Effectiveness Ratios
WTP: Willingness-To-Pay
PSA: Probabilistic Sensitivity Analyses
AI: Artificial Intelligence
IARC: International Agency for Research on Cancer
DCIS: Ductal Carcinoma in Situ
TDABC: Time-Driven, Activity-Based Costing

## References

[1] World Health Organization. Breast Cancer Fact Sheet. 2022. Available from: https://www.who.int/news-room/fact-sheets/detail/breast-cancer?utm_source=chatgpt.com. Cited 2025 Aug 19.

[2] Soleimani M, et al. Exploring the geospatial epidemiology of breast cancer in Iran: identifying significant risk factors and spatial patterns for evidence-based prevention strategies. BMC Cancer. 2023;23(1):1219. 10.1186/s12885-023-11555-1. 38082251 10.1186/s12885-023-11555-1 PMC10712175

[3] Haghpanah S, et al. Investigating the trends of incidence rates of breast cancer in Southern Iran: a population based survey. BMC Womens Health. 2023;23(1):589. 10.1186/s12905-023-02757-7. 37950182 10.1186/s12905-023-02757-7 PMC10638837

[4] International Agency for Research on Cancer. Cancer in Iran: Projections and Trends. 2022. Available from: https://www.iarc.who.int/news-events/cancer-in-iran-2008-to-2025-recent-incidence-trends-and-short-term-predictions-of-the-future-burden/?utm_source=chatgpt.com. Cited 2025 Aug 19.

[5] Wilkinson AN, et al. Cost-Effectiveness of Breast Cancer Screening Using Digital Mammography in Canada. JAMA Netw Open. 2025;8(1):e2452821. 10.1001/jamanetworkopen.2024.52821. 39745700 10.1001/jamanetworkopen.2024.52821 PMC11696453

[6] Akbari ME, et al. Breast cancer status in Iran: Statistical analysis of 3010 cases between 1998 and 2014. Int J breast cancer. 2017;2017(1):2481021. 29201466 10.1155/2017/2481021 PMC5671722

[7] Briggs A, Sculpher M, Claxton K. Decision modelling for health economic evaluation. Oup Oxford; 2006.

[8] Drummond MF, et al. Methods for the economic evaluation of health care programmes. Oxford University Press; 2015.

[9] Organization WH. Data visualization tools for exploring the global cancer burden in 2022. 2022. Available from: https://gco.iarc.fr/today/en. Cited 2025 Aug 19.

[10] Organization WH. Life tables by country Iran. Available from: https://apps.who.int/gho/data/view.searo.60760?lang=en. Cited 2025 Jan 7.

[11] Afsharfard A, et al. Trends in epidemiology, clinical and histopathological characteristics of breast cancer in Iran: results of a 17 year study. Asian Pac J Cancer Prev. 2013;14(11):6905–11. 10.7314/apjcp.2013.14.11.6905. 24377624 10.7314/apjcp.2013.14.11.6905

[12] Harirchi I, et al. Twenty years of breast cancer in Iran: downstaging without a formal screening program. Ann Oncol. 2011;22(1):93–7. 10.1093/annonc/mdq303. 20534622 10.1093/annonc/mdq303

[13] Harirchi I, et al. Decreasing trend of tumor size and downstaging in breast cancer in Iran: results of a 15-year study. Eur J Cancer Prev. 2010;19(2):126–30. 10.1097/CEJ.0b013e328333d0b3. 19952761 10.1097/CEJ.0b013e328333d0b3

[14] Salim M, et al. External evaluation of 3 commercial artificial intelligence algorithms for independent assessment of screening mammograms. JAMA Oncol. 2020;6(10):1581–8. 32852536 10.1001/jamaoncol.2020.3321 PMC7453345

[15] Okonkwo QL, et al. Breast cancer screening policies in developing countries: a cost-effectiveness analysis for India. JNCI: J Natl Cancer Inst. 2008;100(18):1290–300. 18780864 10.1093/jnci/djn292

[16] Abedi G, et al. Survival Rate of Breast Cancer in Iran: A Meta-Analysis. Asian Pac J Cancer Prev. 2016;17(10):4615–21. 10.22034/apjcp.2016.17.10.4615. 27892673 10.22034/APJCP.2016.17.10.4615 PMC5454606

[17] Nemati S, et al. National surveillance of cancer survival in Iran (IRANCANSURV): Analysis of data of 15 cancer sites from nine population-based cancer registries. Int J Cancer. 2022;151(12):2128–35. 35869869 10.1002/ijc.34224

[18] Zahl PH, Maehlen J, Welch HG. The natural history of invasive breast cancers detected by screening mammography. Arch Intern Med. 2008;168(21):2311–6. 10.1001/archinte.168.21.2311. 19029493 10.1001/archinte.168.21.2311

[19] Duffy SW, et al. Markov models of breast tumor progression: some age-specific results. JNCI Monogr. 1997;1997(22):93–7. 10.1093/jncimono/1997.22.93 9709283

[20] Tan SY, et al. Chap. 9: *the MISCAN-Fadia continuous tumor growth model for breast cancer*. JNCI Monogr. 2006;2006(36):56–65. 10.1093/jncimonographs/lgj009 17032895

[21] Tahmasebi S, et al. Chronological changes and trend of breast cancer clinics and pathology among Iranian women during 22 years from the largest breast cancer registry in Iran. World J Surg Oncol. 2019;17:1–7. 31801561 10.1186/s12957-019-1757-7 PMC6894255

[22] Emrani Z, et al. Health-related quality of life measured using the EQ-5D–5 L: population norms for the capital of Iran. Health Qual Life Outcomes. 2020;18:1–8. 32334604 10.1186/s12955-020-01365-5 PMC7183694

[23] Shi J, et al. Cost-effectiveness evaluation of risk-based breast cancer screening in Urban Hebei Province. Sci Rep. 2023;13(1):3370. 36849794 10.1038/s41598-023-29985-z PMC9971026

[24] Jalali FS, et al. Economic burden of breast cancer: a case of Southern Iran. Cost Eff Resource Allocation. 2023;21(1):58. 10.1186/s12962-023-00470-8 PMC10466748 37644546

[25] Wilkinson AN, et al. Capturing the true cost of breast cancer treatment: molecular subtype and stage-specific per-case activity-based costing. Curr Oncol. 2023;30(9):7860–73. 37754486 10.3390/curroncol30090571 PMC10527628

[26] Reddy SR, et al. Cost of cancer management by stage at diagnosis among Medicare beneficiaries. Curr Med Res Opin. 2022;38(8):1285–94. 35285354 10.1080/03007995.2022.2047536

[27] van Ravesteyn NT, et al. Prediction of higher mortality reduction for the UK Breast Screening Frequency Trial: a model-based approach on screening intervals. Br J Cancer. 2011;105(7):1082–8. 21863031 10.1038/bjc.2011.300 PMC3185930

[28] Ahern C, et al. Cost-effectiveness of alternative strategies for integrating MRI into breast cancer screening for women at high risk. Br J Cancer. 2014;111(8):1542–51. 25137022 10.1038/bjc.2014.458 PMC4200098

[29] Hill H, et al. The cost-effectiveness of risk-stratified breast cancer screening in the UK. Br J Cancer. 2023;129(11):1801–9. 37848734 10.1038/s41416-023-02461-1 PMC10667489

